# Digital Mapping of Soil Organic Carbon Based on Machine Learning and Regression Kriging

**DOI:** 10.3390/s22228997

**Published:** 2022-11-21

**Authors:** Changda Zhu, Yuchen Wei, Fubin Zhu, Wenhao Lu, Zihan Fang, Zhaofu Li, Jianjun Pan

**Affiliations:** 1 College of Resources and Environmental Sciences, Nanjing Agricultural University, No. 1 Weigang, Xuanwu District, Nanjing 210095, China; 2 College of Resources and Environmental Sciences, Zijingang Campus, Zhejiang University, 866 Yuhangtang Road, Xihu District, Hangzhou 310058, China

**Keywords:** digital soil mapping, SOC, soil genesis, machine learning (ML), ensemble model, regression kriging (RK)

## Abstract

In the last two decades, machine learning (ML) methods have been widely used in digital soil mapping (DSM), but the regression kriging (RK) model which combines the advantages of the ML and kriging methods has rarely been used in DSM. In addition, due to the limitation of a single-model structure, many ML methods have poor prediction accuracy in undulating terrain areas. In this study, we collected the SOC content of 115 soil samples in a hilly farming area with continuous undulating terrain. According to the theory of soil-forming factors in pedogenesis, we selected 10 topographic indices, 7 vegetation indices, and 2 soil indices as environmental covariates, and according to the law of geographical similarity, we used ML and RK methods to mine the relationship between SOC and environmental covariates to predict the SOC content. Four ensemble models—random forest (RF), Cubist, stochastic gradient boosting (SGB), and Bayesian regularized neural networks (BRNNs)—were used to fit the trend of SOC content, and the simple kriging (SK) method was used to interpolate the residuals of the ensemble models, and then the SOC and residual were superimposed to obtain the RK prediction result. Moreover, the 115 samples were divided into calibration and validation sets at a ratio of 80%, and the tenfold cross-validation method was used to fit the optimal parameters of the model. From the results of four ensemble models: RF performed best in the calibration set (R^2^_c_ = 0.834) but poorly in the validation set (R^2^_v_ = 0.362); Cubist had good accuracy and stability in both the calibration and validation sets (R^2^_c_ = 0.693 and R^2^_v_ = 0.445); SGB performed poorly (R^2^_c_ = 0.430 and R^2^_v_ = 0.336); and BRNN had the lowest accuracy (R^2^_c_ = 0.323 and R^2^_v_ = 0.282). The results showed that the R^2^ of the four RK models in the validation set were 0.718, 0.674, 0.724, and 0.625, respectively. Compared with the ensemble models without superimposed residuals, the prediction accuracy was improved by 0.356, 0.229, 0.388, and 0.343, respectively. In conclusion, Cubist has high prediction accuracy and generalization ability in areas with complex topography, and the RK model can make full use of trends and spatial structural factors that are not easy to mine by ML models, which can effectively improve the prediction accuracy. This provides a reference for soil survey and digital mapping in complex terrain areas.

## 1. Introduction

The soil sphere is an important component of the earth’s ecosystem and a major site for human subsistence and social production activities. The soil carbon pool is the largest terrestrial organic carbon pool, and it plays an important role in the global carbon cycle and maintaining the ecological balance in the context of climate warming and land use change [1]. Soil surveying and mapping are prerequisites for better utilization and management of limited soil resources, and they are an important foundation for other soil science research. Traditional soil mapping is carried out by experts through field surveys to understand soil–landscape relationships and manual mapping based on topographic maps and aerial or satellite imagery [2]. The traditional method is not only time-consuming and laborious but also relies heavily on expert knowledge and is difficult to apply to soil investigations on a large scale or outside the study area. With the development and application of soil genesis theory and the law of geographical similarity, digital soil mapping (DSM) has emerged [3,4,5]. DSM predicts soil properties or types by mining the relationship between soil and environmental covariates through machine learning (ML) and other methods [6]. It can save a lot of labor and time costs and is free from the limitation of expert knowledge limited to a certain area.

Soil genesis theory is the theoretical basis for DSM, which states that soils are formed through certain physical, chemical, and biological processes under the action of five soil-forming factors: climate, topography, parent material, biology, and time [7]. Soil is an object with specific properties formed under specific environmental conditions, and the spatial distribution of soil has a synergistic relationship with the spatial distribution of environmental factors [8]. Soil and environmental factors are geographically similar, and the more similar the combination of environmental factors is, the more similar the corresponding soil is. The environmental factors and soil properties are mostly continuous and gradual, and the closer the distance is, the closer the soil properties are, that is, the first law of geography [9]. This geographic similarity is another theoretical basis for DSM. Based on the above theories, McBratney [8] proposed a soil spatial prediction function with spatially autocorrelated errors (SSPFe), which is particularly relevant for those places where soil resource information is limited.

Kriging interpolation is an early method for DSM [10]; the geostatistical approach treats soil properties and environmental covariates as regionalized variables and expresses the spatial correlation of variables as differences in distance and direction. However, the geostatistical method is based on spatial point data for interpolation and cannot fully utilize the surface data of environmental covariates (e.g., remotely sensed images, DEM, and their derived variables). ML and data mining methods, on the other hand, can solve this problem by bringing the spatial surface raster data of environmental variables into the fitted model for soil prediction mapping. In recent years, ML methods have been used to conduct many DSM studies, such as multiple linear regression (MLR), random forest (RF), regression trees (RT), artificial neural network (ANN), and Cubist [11,12,13,14,15,16,17,18]. However, most of these methods use a single model or an ensemble model of the same structure, which is limited by the single-model structure and has a low prediction accuracy in the complex areas with undulating terrain. In addition, there are also scholars who combine ML with expert knowledge for soil mapping, such as the soil land inference model (SoLIM) [19] and high-accuracy surface modeling (HASM) [20,21]. HASM assumes that soil properties are constant within the same type of area, and it was first used in DSM to predict soil pH [22], but this method may not be applicable in areas where soil properties are often asymptotic due to less human influence, and a large number of samples are required to calculate the average value for each partition.

Soil is formed under the combined action of multiple environmental factors, and it is difficult for the modeling process to take all the environmental factors into account. Limitations on the type of input environmental covariates, as well as the influence of the model structure and parameter selection, can prevent the model from fully exploiting the relationship between soil and environmental factors and can lead to a certain amount of model error [23]. The error is manifested as the residuals between the predicted and actual measured values of the fitted model, and the residuals have some structural and spatial autocorrelation as environmental variables. Therefore, the residuals can be interpolated by geostatistical methods, and the spatial variation pattern of the residuals is attributed to a function of distance and direction. Combining regression models and geostatistics, the regression kriging (RK) method can not only make full use of the spatial surface data of environmental covariates to improve the prediction accuracy but also reduce the error of the model through residual interpolation with spatial autocorrelation. Although the RK method has the advantages of both ML and geostatistics, they have been less studied in DSM [4].

The main purpose of this study was to compare the accuracy and potential of the ML and the RK models for SOC mapping in undulating complex terrain areas. Four ML methods—RF, Cubist, stochastic gradient boosting (SGB), and Bayesian regularized neural networks (BRNNs)—were used to fit the trend between SOC and environmental covariates, and simple kriging (SK) was used to spatially interpolate the residuals of each sampling point. We then performed residual correction on the prediction results of the ML models to obtain the final SOC prediction map.

## 2. Materials and Methods

### 2.1. Study Area

The study area was a hilly farming area in the Ningzhen mountain range in the middle and lower reaches of the Yangtze River, which is located in the north area of Jurong City, Jiangsu Province of China. It is located in a warm temperate continental monsoon climate zone, where rice and maize are grown in summer and wheat and rape are mostly grown in winter. In addition, there are a few natural woodlands and artificial gardens. The terrain of the study area was complex and undulating, with an altitude of 71–145 m a.s.l.

**Figure 1:**
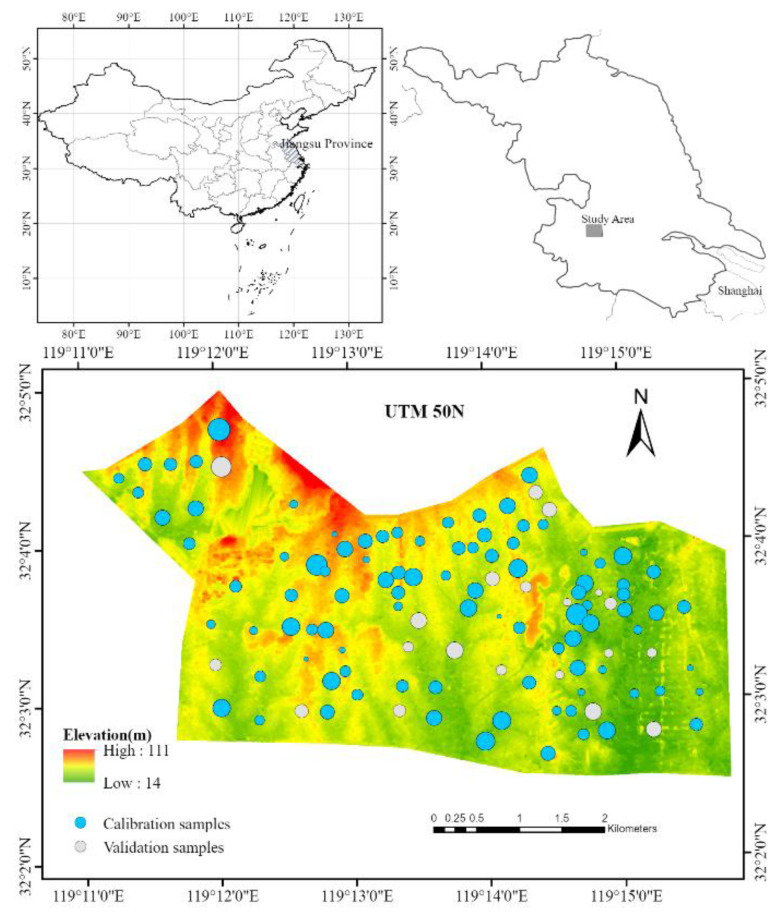
Distribution of study area and sampling points. Note: the sample point size represents the amount of SOC content (4.29~19.79 g/kg).

Based on the principles of uniformity and representativeness, 115 soil samples were laid out and collected from May to June 2021 using satellite images and topographic maps of the study area as auxiliary information. The distribution of the study area and sampling points is shown in Figure 1. At each sampling point, typical plots were selected, and the five-point sampling method was used to conduct mixed sampling of the surface (0–20 cm) soil. The coordinates and elevation of each sample point were recorded by GPS, and the landscape features such as topography and vegetation around the sample point were also recorded. These samples were brought back to the laboratory and sieved by natural air-drying and grinding, and then the SOC content of each sample was determined by oxidation of sulfuric acid–potassium dichromate solution with external heating.

### 2.2. Predictor Variables

The theory of soil genesis holds that soil is formed under five soil-forming factors: climate, parent material, topography, biology, and time, which provide a good theoretical support for DSM [7]. The factors that are dominant, stable, and easily accessible are selected as predictor variables according to the role of various soil-forming factors in the soil genesis process. All of the study area had a warm temperate continental monsoon climate, and the variability in temperature, precipitation, and parent material was weak, so these factors were not suitable to be used as predictor variables. Temporal factors of soil are often difficult to express and not easily accessible, and they are not used as predictor variables because they are eventually expressed as differences in soil property values or types in the soil formation process. Therefore, topographic indices were extracted as topographic factors based on DEM, and vegetation indices and soil indices were extracted as biological factors and soil factors, based on remote sensing images, as the input predictor variables. We calculated 10 topographic indices, 7 vegetation indices, and 2 soil indices, which follow in Section 2.2.1 and Section 2.2.2, and the formulas of several uncommon indices are listed in Table 1.

#### 2.2.1. DEM and Topographic Indices

China Resource-3 02 satellites carry a three-line array mapping camera and a multispectral camera, and their mapping camera can generate 2.1 m resolution forward-looking panchromatic images and 2.5 m resolution forward-looking and back-looking 22° panchromatic images. We purchased and downloaded the ZY-3 02 image of 15 November 2019 from China Center for Resources Satellite Data and Application (CRESDA, https://data.cresda.cn/#/home). Ten DEM-derived terrain indices were calculated in SAGA (System for Automated Geoscientific Analysis, http://www.saga-gis.org) v. 8.1.3 [24,25], including channel network base level (CNBL), channel network distance (CND), convergence index (CI), plan curvature (PlC), profile curvature (PrC), slope, topographic wetness index (TWI), valley depth (VD), relative slope position (RSP), and length–slope factor (LSF).

#### 2.2.2. Image Processing and Spectral Indices

The image data used for modeling were obtained from European Space Agency (ESA) Open Access Hub (copernicus.eu). Sentinel-2A and 2B binary stars have a replay period of 5 days with high temporal and spatial resolution (10 m resolution in 4 visible and near-infrared bands). In this study, the Sentinel-2B (Level-1C product) image of 31 August 2021 was downloaded as the source data by selecting images with low cloudiness during the crop flowering–irrigation period (late August to late September). Atmospheric correction was performed in Sen2Cor to generate Level-2A product, and the image covering the study area was resampled to 10 m and cropped in SNAP. Seven bands of S2B were selected to export to ENVI format for subsequent operations, including B2, B3, B4, B8, B8A, B11, and B12. Because of the close relationship between vegetation growth and SOC content, seven vegetation indices were calculated in ENVI 5.3 [14,15], including ratio vegetation index (RVI), difference vegetation index (DVI), normalized difference vegetation index (NDVI), enhanced vegetation index (EVI), soil-adjusted vegetation index (SAVI), modified soil-adjusted vegetation index (MSAVI), and soil-adjusted total vegetation index (SATVI). In addition, two soil indices, soil color index (SCI) and soil red index (SRI), were calculated as predictor variables to reflect properties such as soil color and hematite [26].

### 2.3. Trend Models and RK Modeling Technique

The four ensemble models and RK model for SOC content prediction mapping are described in this section. The RK model includes two parts: trend prediction and residual prediction. The topographic and spectral indices (vegetation and soil indices) corresponding to the soil sample points were extracted in ArcGIS 10.8 as predictor variables, and the SOC content was used as the response variable for modeling. The ensemble model is constructed by ensemble learning or the ensemble method, which integrates multiple learners or algorithms. Four ensemble models, including RF, Cubist, SGB, and BRNN, were used for trend analysis in R 4.1.2, and SK was used for residual analysis in ArcGIS. The results of the two were superimposed to obtain the final RK prediction result. Four trend models were established through the “ranger”, “cubist”, “gbm”, and “brnn” functions of the “caret” package in R, then parameter tuning and optimal model selection were performed.

#### 2.3.1. Random Forest

RF is an ensemble model based on the bagging algorithm. RF uses the decision tree as the base classifier and the bootstrap method for sampling of put-back, and n models (base learner) were trained to synthesize the results of n models for judgment, thus effectively avoiding the problem of overfitting of individual models [27]. In addition, due to the use of the “ bootstrap” sampling strategy, the out-of-bag (OOB) error is calculated as a test set when the model is not sampled during training, so there is no need to carry out cross-validation separately. The “ranger” function in the “caret” package is called in R for random forest prediction, and two parameters need to be defined: (i) mtry: the number of randomly selected predictor variables at each node; (ii) min.node.size: the minimum number of nodes, which represents the minimum amount of data for the tree training process. In this study, the tuning grid was set with mtry from 2 to 10 (step size 1) and min.node.size from 5 to 25 (step size 5), and the parameter with the lowest root mean square error (RMSE) from cross-validation was selected for the best modeling.

#### 2.3.2. Cubist

Cubist is a decision tree method developed from the M5 model tree [28], similar to a stacking algorithm that combines decision trees and multiple linear regression methods, where each data subregion corresponds to a different segmented linear model, integrating multiple segmented linear models and tree models [29]. Compared to the simple CART leaf node on which there is a specific value, and the Cubist leaf node which is a linear regression model, the nonlinear problem can be well solved by a series of combined segmented linear models [30]. In addition, the method has the function of variable importance analysis, and RF also has this function. In this study, the “Cubist” package and the “caret” package were combined for regression modeling, and optimization was carried out through two parameters: committees and neighbors. Committees were set from 5 to 50 (step size 5) and neighbors were set to (1, 5, 9) for iterations to determine the optimal model.

#### 2.3.3. Stochastic Gradient Boosting

Gradient boosting machine (GBM) is an ensemble model based on the idea of boosting, which can reduce the variance and bias of individual models to improve the fitting accuracy [31]. The base learner is first built to explain the residuals and reduce them in the gradient direction to find a model that minimizes the expectation of the loss function by iterations. The use of a resampling method in building the base learner is called stochastic gradient boosting (SGB) [32]. In this study, SGB was modeled by the “gbm” function in the “caret” package, which has four parameters: (i) the number of iterations (n.trees); (ii) the learning efficiency (shrinkage); (iii) the depth of the decision tree (interaction.depth); (iv) the minimum number of nodes (n.minobsinnode). The depth of the decision tree was set to (1, 5, 9), the minimum number of nodes was set to (10, 15, 20), the number of iterations was set from 50 to 3000 (step size 50), and the learning efficiency was set from 0.001 to 0.01 (step size 0.001) with the tuning grid of learning efficiency for parameter tuning.

#### 2.3.4. Bayesian Regularized Neural Networks

The BRNN model adds regularization coefficients (penalty terms) to the conventional neural network to balance the contradiction between the model prediction accuracy and generalization ability; thus, it can reduce the overfitting phenomenon by correcting the training performance function of the network [33]. In this study, model fitting was performed by the “brnn” function in the “caret” package. The main parameter is the number of neurons in the hidden layer, and the number of neurons was set from 1 to 20 (step size 1) for iterative determination of the optimal.

#### 2.3.5. RK Modeling Technique

Based on the above theories, McBratney proposed a soil spatial prediction function with spatially autocorrelated errors (SSPFe) (Equation (1)) [8].

(1) $$S_{a}\;or\;S_{c}=f\left(s,\;c,\;o,\;r,\;p,\;a,\;n\right)+e,$$

where $S_{a}\;or\;S_{c}$ represents soil properties or soil types; s represents other soil information at the same point; c represents climatic factors such as temperature and precipitation; o represents biological factors such as fauna, vegetation cover, and anthropogenic use; r represents topographic and geomorphological features; p represents soil parent material or lithological characteristics; a represents the time factor of soil formation; n represents spatial location; and e represents the residuals with spatial autocorrelation.

The spatial distribution trends of soils can be described by a trend fitting model based on environmental covariates and a spatial interpolation based on the residuals of the trend model, which constitute the main approach of the RK model [34] that can be expressed as Equation (2):

(2) $$\hat{z}\left(s_{0}\right)=\hat{m}\left(s_{0}\right)+\hat{e}\left(s_{0}\right)=f_{s_{0}}\left(q_{k}\left(s_{0}\right)\right)+\sum_{i=1}^{n}\lambda_{i}\cdot e\left(s_{i}\right),\;k=1,2,\ldots ,p;\;i=1,2,\ldots ,n,\;$$

where $\hat{z}\left(s_{0}\right)$, $\hat{m}\left(s_{0}\right)$, $\hat{e}\left(s_{0}\right)$ refer to the RK prediction value, the fitted drift, and the interpolated residual at point $s_{0}$, respectively, p refers to the number of environmental covariates, n refers to the number of sampling points, $q_{k}\left(s_{0}\right)$ refers to the number of environmental covariates at point $s_{0}$, $f_{s_{0}}\left(q_{k}\left(s_{0}\right)\right)$ refers to the functional relationship between soil and environmental covariates $q_{k}$ at the point $s_{0}$, $\lambda_{i}$ refers to kriging weights determined by the spatial dependence structure of the residual, and $e\left(s_{i}\right)$ refers to the residual at the sampling point $s_{i}$.

Due to the expectation of the residuals being fixed, the SK method can be applied to the residuals of the model [8]. The lag size and the number of lags are the two most important parameters of kriging interpolation, which have a great impact on the interpolation accuracy in the region. The average nearest neighbor (ANN) method can be used in ArcGIS to calculate the average minimum interval between sample points and neighboring elements as the step size, and the step interval can be reduced appropriately to amplify the local variation details between neighboring samples in the area of obvious aggregation of sample point space. Then, through cross-validation, the parameters with the RMSE minimum can be selected for optimal modeling.

### 2.4. Model Performance Evaluation

In this study, three evaluation metrics were calculated to analyze the model performance: RMSE, R^2^, and MAE. The RMSE was used to measure the accuracy of the model, and the smaller the RMSE, the higher the accuracy of the model. The R^2^ was used to verify the stability of the model, and the larger the R^2^, the more stable the model. The sample set was divided into a calibration set and validation set by 80%, and the fitting accuracy of the calibration set and validation set were expressed as RMSEc and RMSEv, respectively. These validation metrics are calculated as follows:

(3) $$RMSE=\sqrt{\frac{1}{n}\sum_{i=1}^{n}{\left({\hat{y}}_{i}-y_{i}\right)}^{2}},\;$$

(4) $$R^{2}=\sum_{i=1}^{n}{\left({\hat{y}}_{i}-\bar{y_{i}}\right)}^{2}/\sum_{i=1}^{n}{\left(y_{i}-\bar{y_{i}}\right)}^{2},\;$$

(5) $$MAE=\frac{1}{n}\sum_{i=1}^{n}\left|{\hat{y}}_{i}-y_{i}\right|,\;$$

where n represents the number of sample points, and $y_{i}$ and ${\hat{y}}_{i}$ represent the observed and predicted values of SOC content of site i, respectively.

## 3. Results and Analysis

### 3.1. Data Preprocessing and Exploratory Analysis

**Figure 2:**
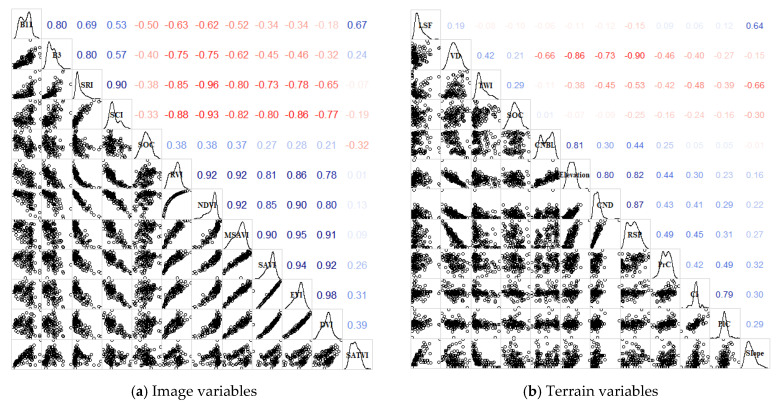
Variable Pearson correlation coefficient matrix. Notes: The diagonal line indicates the variable names and distribution density curves, the upper diagonal panel is the correlation coefficient matrix, and the lower diagonal panel is the scatter plot matrix between the variables. SCI, soil color index; SRI, soil red index. RVI, ratio vegetation index; DVI, difference vegetation index; NDVI, normalized difference vegetation index; EVI, enhanced vegetation index; SAVI, soil-adjusted vegetation index; MSAVI, modified soil-adjusted vegetation index; SATVI, soil-adjusted total vegetation index. CNBL, channel network base level; CND, channel network distance; CI, convergence index; PlC, plan curvature; PrC, profile curvature; TWI, topographic wetness index; VD, valley depth; RSP, relative slope position; LSF, length–slope factor.

The feature variables with a high correlation with SOC were selected according to the correlation test, the linear correlation between the variables was tested by Pearson correlation coefficient, and the features were ranked in importance according to the principal component analysis (PCA) method (Figure 2). The scatter plot in the lower left panel of the correlation matrix shows the trend of the fit between the variables. The nonlinear correlation was tested by the distance correlation coefficient (Table 2). Although the correlation between the topographic index and SOC is lower than that of the vegetation index and SOC, the topographic index is mainly affected by DEM accuracy and raster scale, whereas it is less affected by human factors and environmental factors, and it has a long time stability which can effectively improve the model accuracy and stability, so it is necessary to use the topographic index with a certain correlation with SOC as the input variable. The variables with a correlation greater than 0.2 with SOC were selected in the image-derived variables, the variables with a correlation greater than 0.15 with SOC were selected in the DEM-derived variables, and the feature variables with covariance were excluded according to the threshold value of a correlation greater than 0.85 in absolute value. In the end, five vegetation and soil factors (RVI, NDVI, SATV, SCI, and SRI) and five topographic factors (Slope, TWI, RSP, CI, and PrC) were selected as predictor variables.

The 115-sample data set was partitioned into a calibration set and a validation set with 95 and 20 sample points each at an 80% ratio. Descriptive statistics (Table 3) were performed for the calibration and validation sets, and the *p*-values > 0.05 by the K-W normality test were consistent with a normal distribution, which is also evident from the overall SOC distribution density curve on the diagonal of Figure 2. The coefficient of variation, skewness, and kurtosis values of the calibration set is very close to those of the total set data, which indicates that the calibration set is well representative of the overall sample. The independent variables in the calibration and validation sets were preprocessed in R, and the ten environmental covariates were tested for covariance and approximate zero variance, and then centralized and standardized to prevent the covariance of the features and the different data outline from affecting the instability of the model.

### 3.2. Performance Analysis of Trend Models

**Figure 3:**
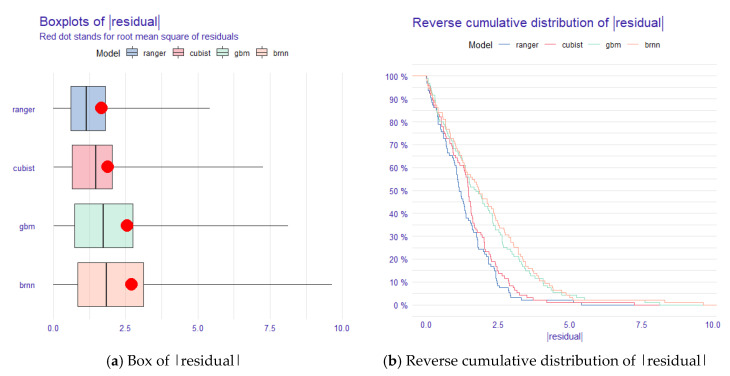
Absolute residuals and reverse cumulative distribution curves of absolute residuals for the ranger, Cubist, GBM, and BRNN models.

In order to evaluate the performance of the trend-fit models, explanatory analyses were performed for each of the four models after determining the best-fit parameters, and the results are shown in Figure 3.

From the analysis in the box plot of model residuals (Figure 3a), we can conclude that the average absolute residuals of ranger and Cubist are low, and the overall distribution is at a low level, but the maximum value of the absolute residuals of Cubist is slightly higher, indicating that the difference between its predicted maximum or minimum value and the actual value of SOC is large. While BRNN has the largest absolute residual, GBM is second, and the average absolute residuals of both GBM and BRNN are high, indicating that all the predicted values of these two models differ from the real values of SOC. In the plot of the reverse cumulative distribution of absolute residuals (Figure 3b), it can be concluded that the cumulative values of ranger and Cubist residuals increase slowly with the increase in modeling sample data, and the model performance is more stable, while the cumulative curves of GBM and BRNN residuals increase faster after the input sample reaches 45%, and the model performance is not stable enough. Overall, the ranger model has the highest accuracy and better stability in the model performance of the calibration set, followed by the Cubist model, while the BRNN model has the lowest accuracy and the worst stability, followed by the GBM model.

### 3.3. Residual Analysis and Geostatistical Modeling

**Figure 4:**
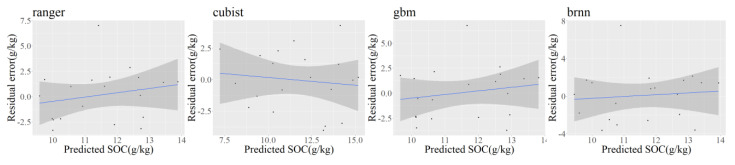
Residual–predicted value fit plot of the ML models: ranger, Cubist, GBM, and BRNN. Note: the shaded area indicates the confidence interval of the fitted line at the 95-percent confidence level.

Before applying geostatistics to the residual, we should check whether there is an obvious trend between the predictor and target variables in the ML models according to the residual–predicted value fit plot (Figure 4). The analysis showed that the four ML models have almost no trend, and the fitted curves are close to horizontal, achieving very good fitting results. The soil samples corresponding to the extreme values of residuals were all from natural woodland. The number of samples from woodland was much smaller than that from agricultural land due to the small area of natural woodland in the study area, while the SOC content of natural woodland was generally much higher than that of agricultural land, which might cause the model to fit most of the sample points of agricultural land and result in larger residuals of woodland sample points. Overall, the residuals and SOC predicted values in the residual–predicted scatter plot showed a random distribution with no obvious trend, indicating that the relationship between the target and predicted variables was almost fully expressed by the model.

**Figure 5:**
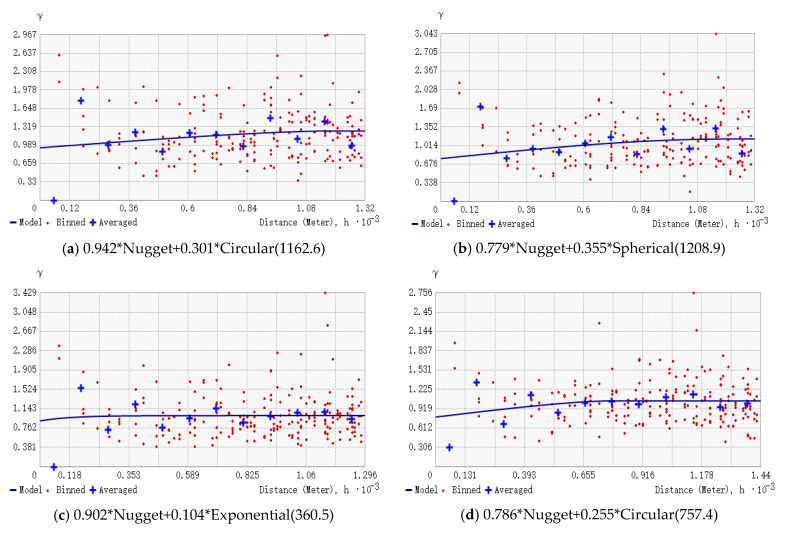
Semi-variance function and main parameters of the residuals of the ML models: (**a**) rangerresidual error, (**b**) Cubist residual error, (**c**) GBM residual error, (**d**) BRNN residual error.

The four models tapped most of the correlations between the target variables and the environmental covariates, but the residuals still have some spatial autocorrelation due to the influence of other structural environmental factors. SK was selected for geostatistical analysis in ArcGIS, and we set the appropriate step size and number of steps by observing the change trend of the residual semi-variogram value with distance. The semi-variogram function of the residuals of the four models is shown in Figure 5. The spatial autocorrelation of all four model residuals is low, and the block gold value is high, close to the pure block gold effect, which reflects the success of the trend models’ fitting.

### 3.4. Evaluation and Comparison of Trend and RK Models

In order to evaluate the performance of the residual kriging interpolation in different trend models, the residuals of the four trend models were interpolated separately, and the trend model predictions and residual kriging predictions at the same location were extracted and superimposed based on the sample points. At the same time, the model accuracy was tested with the test set to assess the generalization ability of the models, and the results are shown in Table 4 (the models with the highest accuracy are marked in bold, and the models with the second highest accuracy are marked in bold italics).

By comparing the results of the four trend models, it was concluded that the ranger model had the highest accuracy and the lowest error in the calibration set (R^2^c = 0.834, RMSEc = 1.669 g/kg) and slightly lower accuracy in the test set (R^2^v = 0.362, RMSEv = 2.493 g/kg), but the Cubist model performed well in both the calibration and validation sets (R^2^c = 0.693, RMSEc = 1.880 g/kg and R^2^v = 0.445, RMSEv = 2.283 g/kg). However, the BRNN model performed the worst in both the calibration and validation sets, and the GBM model had the next highest accuracy, with the BRNN model explaining only about 30% of the variation (R^2^c = 0.323, RMSEc = 2.712 g/kg and R^2^v = 0.282, RMSEv = 2.589 g/kg) and the GBM model explaining about 40% of the variation (R^2^c = 0.430, RMSEc = 2.551 g/kg and R^2^v = 0.336, RMSEv = 2.522 g/kg). On the other hand, by comparing the performance of the four RK models, it can be seen that the trend models superimposed with residual kriging interpolation can greatly improve the prediction ability of the model, especially in the test set. The prediction accuracy of the four RK models reached a high level, with the prediction accuracy R^2^ greater than 0.56 in the calibration set and R^2^ greater than 0.62 in the test set. Moreover, in the comparison with the trend model without residual interpolation, the prediction accuracy of the four RK models improved by 0.033, 0.101, 0.304, and 0.246 in the calibration set, and by 0.356, 0.229, 0.388, and 0.343 in the test set, respectively. Due to the fact that the Cubist model predictions are closer to the true values (the predicted–observed fit line is close to 1:1), the R^2^ of the Cubist model is not the highest, but the RMSE is the smallest, and the smaller residual value domain of the trend model makes the overall residuals of the RK-Cubist model small.

**Figure 6:**
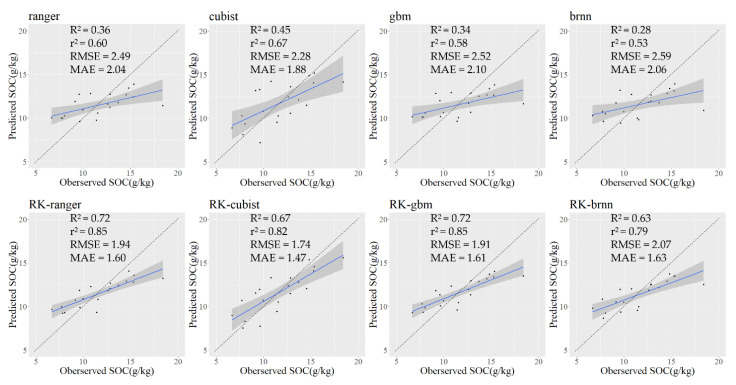
Predicted–observed fitted line of the ML models and RK models. Notes: The shaded area indicates the confidence interval of the fitted line at the 95-percent confidence level. The r^2^ evaluates the deviation from the 1:1 line, whereas the R^2^ evaluates the deviation from the fitted linear regression line between measured and predicted values.

The performance of several fitted models can be visualized in the fit plots of predicted values against observed values of the validation samples (Figure 6). The predicted–observed fit curve of the four RK models are closer to 1:1 than that of the trend models, and the scatter plot distribution is more concentrated near the fit curve. In addition, the slope of the predicted–observed fit curve of the RK-GBM and RK-BRNN models increases significantly, indicating that the prediction accuracy has been greatly improved; the slope of the predicted–observed fit curve of the RK-ranger and RK-Cubist models changes less, mainly reflecting that the scatter distribution becomes more concentrated and the scatter points are closer to the fit curve.

### 3.5. Variable Importance Analysis of Different Models

**Figure 7:**
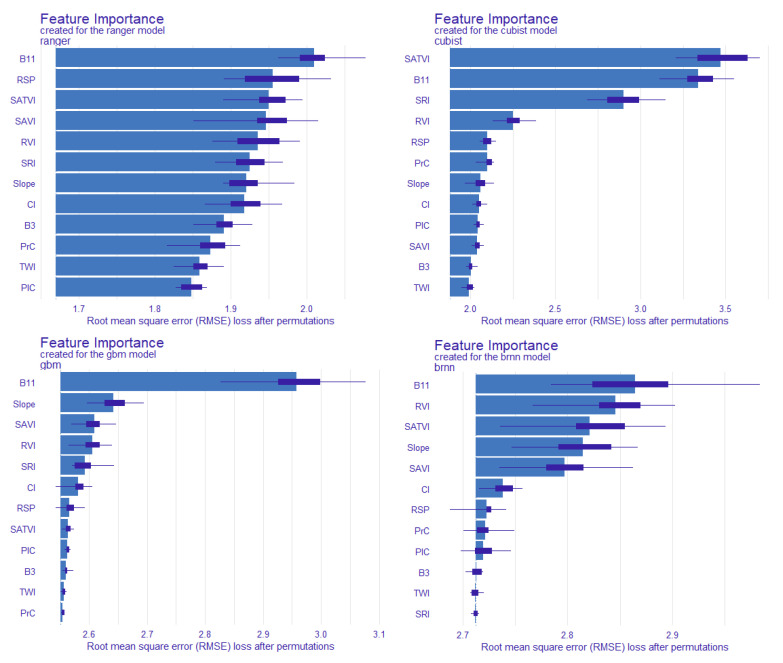
Permutation base on variable importance measures for the explanatory variables. Notes: the box plot indicates the error values of 10 permutations, and the height of bars indicates the mean error values of 10 permutations.

We chose the RMSE as the model performance index to rank the importance of the variables, and, considering the uncertainty of the permutations, we computed the mean values over a set of 10 permutations (Figure 7). For the ranger model, the importance of each predictor variable did not differ significantly, except for the most important, B11; the lack of one of the other variables would increase the RMSE of the model by no more than 0.3 g/kg, and the absolute RMSE was not greater than 2 g/kg. For the Cubist model, the first three variables were more important, namely, SATVI, B11, and SRI, and the remaining variables had low importance. For the GBM model, the importance of the B11 was largest, the importance of the remaining variables was relatively small, and the importance of the last six variables was weak. For the BRNN model, the first five variables had high importance, and the last six variables had a weak impact on the model. In general, the ranger model has the lowest dependence on variables, and the absence of a particular variable does not have a significant impact, whereas the GBM model is too dependent on one variable, which may bring greater instability to the model. Although the importance of the variables varied among the models, B11, RVI, and SATVI were the main explanatory variables for SOC prediction, indicating that the image derivatives were generally more important than the DEM derivatives. This indicates the high relevance and good prediction ability of vegetation and soil indices regarding SOC content.

### 3.6. Spatial Distribution of SOC Content Maps

**Figure 8:**
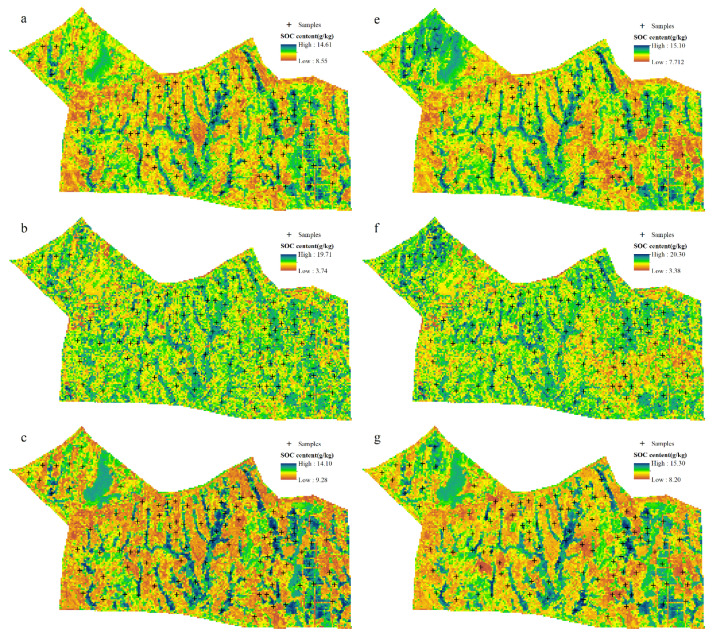

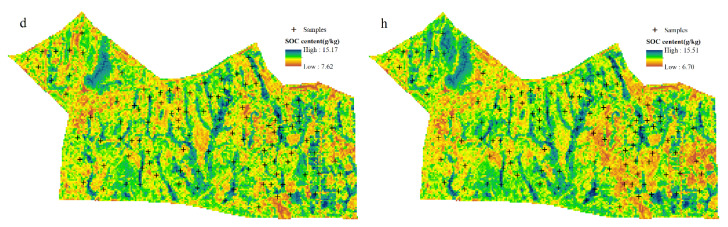
Spatial distribution of SOC predicted by trend models and RK models: (**a**) ranger, (**b**) Cubist, (**c**) GBM, (**d**) BRNN, (**e**) RK-ranger, (**f**) RK-Cubist, (**g**) RK-GBM, (**h**) RK-BRNN.

The optimal parameters of the model were used to predict SOC content, and the spatial distribution maps of SOC content predicted by four trend models and four RK models are presented in Figure 8. The SOC content predicted by the eight models has similar spatial distribution characteristics, with low SOC content at high terrain and high SOC content at low terrain, which indicates an obvious correlation between SOC content and terrain. Topography is not determined by absolute elevation but by relative elevation, so that SOC is highly correlated with topographic indices, such as slope position and slope gradient, but not with absolute elevation. When the topography changes in a complex way, there may be a situation where the elevation is the same but the slope is different, and then using the topographic index instead of elevation as a predictor variable can effectively explain the nonlinear relationship. Moreover, topography type integrates topographic factors such as slope position, slope gradient, slope length, etc., which can be used as a predictive variable for DSM. In addition, areas with high SOC content are mainly distributed in areas with a high vegetation index and low soil index, which indicate that SOC has obvious correlation with soil color, soil mineral composition, and surface vegetation cover.

For the ranger and BRNN models, the SOC content prediction value range is smaller than the observed value range (4.29~19.79 g/kg); the ranger spatial distribution boundary is obvious, but the BRNN spatial distribution boundary is fuzzy. However, the SOC content prediction value range of the Cubist model is the largest (3.74~19.71 g/kg), slightly larger than the observed value range. The predicted SOC content map of the GBM model has obvious distribution boundaries in space, which is not consistent with the asymptotic nature of spatial distribution, and the predicted value range is the smallest (9.28~14.10 g/kg), which is larger than the observed value range of SOC content and also leads to its lower prediction accuracy in the calibration and test sets, which is consistent with the model performance in Table 4.

Due to the inclusion of the effect of residuals, which extends the upper and lower bounds of the predicted values, the predicted value domain of the RK models increases over both the predicted value domains of the corresponding trend models. While the transition boundary of the trend model is more obvious, the RK model weakens this trend, and SOC has better asymptotic variability in space. In the central and eastern parts of the study area, the values became lower in places with high SOC content and higher in places with low SOC content, which reduced the differences between land use types in a small area and was more consistent with the actual distribution. In the natural forest and orchard areas in the northwest, the trend models, in order to fit the whole samples, made the predicted values lower in this area, and the residuals were larger than the observed values. The predicted values of the RK models in the northwestern part of the study area were significantly higher after superimposing the residuals, which improved the fitting accuracy in this area to a large extent.

## 4. Discussion

The reasons affecting the DSM prediction accuracy are mainly composed of three parts: model structure, tuning parameters, and input predictor variables [23]. For the trend models: The RF and Cubist models both have higher model accuracy, and SGB and BRNN have worse accuracy, with high accuracy in the RF calibration set and the Cubist test set. In addition, the prediction value domain of the Cubist model is larger than other models and larger than the upper and lower limits of the observed value domain. This may be due to the fact that RF uses a tree-based learner and the splitting criteria of the tree focus only on the range of relevant features and useful values, which cannot infer higher or lower values than those in the calibration data, and it is almost impossible to obtain values outside the observed interval. In contrast, classical linear regression is less influenced by the range of data values and is good at inferring trends, and the nodes of the Cubist tree model are linear models rather than specific values. The Cubist model combines the advantages of the tree model and the linear model, which make the predicted value domain potentially larger than the observed value domain and has better generalization ability in the unsampled region. In this study, RF as a traditional ensemble model has high prediction accuracy but performs slightly worse in the validation set. Cubist is not only the second most accurate in the calibration set but also has the highest accuracy in the validation set, so we can conclude that Cubist has good prospects for application in SOC prediction in hilly farming areas with complex topography. In addition, all four trend models require user-defined parameters, which can lead to modeling instability and affect their generalization ability and practical applications. To balance the prediction accuracy and stability of the models while ensuring the prediction accuracy, models with the same algorithm but without tuning parameters or with fewer parameters can be selected. For instance, both RF and bagged CART methods are integration models of the tree model and bagging algorithms, but bagged CART can be used with the “treebag“ function without tuning parameters for modeling, which will improve the stability of the model to some extent.

For the RK models: The influence of environmental covariates not incorporated in the trend modeling and the limitations of the model’s structure and parameters can lead to incomplete mining of the relationship between the predictor and the response variables. This relationship, which is not expressed by the trend model, is passed through the model to the residuals of the target variable, causing the residuals to still have a certain trend and exhibit some spatial autocorrelation in space. We can use the kriging method to analyze the residuals of the model with geostatistics, and the remaining trend of the target variable can be tapped to improve the prediction accuracy. It is difficult to achieve a good fitting accuracy with the default parameters of geostatistics, and they often need to be set artificially according to the semi-variance function. The RK method combines the advantages of geostatistics and ML models, which not only exploits the relationship between target variables and known environmental covariates but also makes full use of the spatial autocorrelation of target variables and can effectively reduce the errors caused by uninvolved predictor variables or model instability. The four RK models all improve the prediction accuracy and stability to a great extent, which indicates that the RK method has good scientific research value and application prospects in DSM.

Regarding the improvement of predictor variables and study content: Residuals brought by structural factors with spatial correlation can be well predicted by kriging interpolation, but residuals brought by random factors such as human activities (e.g., land use cover) cannot be expressed. In addition, the residual–predicted value fit plot (Figure 4) analysis shows that the residuals are larger for all sample points in natural woodland, partly because the large area of agricultural land makes the trend model fit the natural woodland sample points less well, and partly because of the non-asymptotic variability between natural woodland and agricultural land, and this abrupt variability can lead to unstable fits. Land use cover has a certain randomness in spatial distribution due to the dual effect of natural and human factors over a long period of time, but it also has a certain dominance and stability in the soil formation process, so it is necessary to fit this change trend of SOC by using land use type as a category factor. From the predicted distribution map of SOC content (Figure 8), the correlation between the spatial distribution of SOC content and terrain height is generally obvious, and the correlation with indices reflecting topographic features such as slope position, slope degree, and moisture index is high, but the correlation coefficient with Jedi elevation is not high. This indicates that there is no obvious relationship between soil properties and elevation in the undulating hilly areas, and topographic indices or relative elevation should be used as predictor variables. In addition, the landscape types related to soil mapping can be studied based on the theory of soil genesis and the law of similarity of the geographic environment, integrating land use types and topographic features, as a way to express the topography, vegetation, and human activities among soil genesis factors, and using the landscape types as key factors to predict soil attributes or types, effectively improving the prediction accuracy and interpretability of DSM.

In general, the machine learning method can make full use of environmental covariate surface data and predict soil properties outside the sampling area, but it needs to be restricted to areas with similar environmental conditions to the study area. In order to improve the generalization ability of the model, it is also necessary to expand the area covered by the study area and increase the environmental complexity of the study. The RK method can offset the effect of residuals with spatial correlation by overlaying residual interpolation and trend model prediction, which can greatly improve the prediction accuracy. However, the kriging method relies on soil sampling point data for interpolation, which cannot be used in unsampled areas, and kriging interpolation results are strongly influenced by the extreme value points, which require a good representation and uniform distribution of sample points. In the case of limited funds or time, or the situation of a large-scale soil survey, RK mapping can be carried out using the sample points of historical soil data.

## 5. Conclusions

In this study, four ensemble models and four RK models were used for SOC mapping. In areas with complex topography, the indices which reflect the topographical features should be used to predict SOC instead of the absolute elevation. Vegetation and soil indices play an important role in predicting SOC, and land use and geomorphic types also have a significant impact on the spatial distribution of soil attributes or types, which should be considered as a key variable for soil mapping.

The Cubist model, due to combining the advantages of linear and tree models, has both high prediction accuracy and generalization ability, and it is more advantageous in studying nonlinear relationships in areas with complex topography. The RK model can make full use of spatial autocorrelation not explained by ML models through geostatistics modeling, so it can largely improve the prediction accuracy of DSM. On the other hand, the kriging method requires that the samples should be evenly distributed and sufficient in number, and the samples of historical legacy soil data can be considered as a supplement in situations of limited research conditions or large-scale soil surveys.

## Figures and Tables

**Table 1:** Calculation formulas of spectral indices.

Indices	Equations	Property
MSAVI	$\frac{2*NIR+1-\sqrt{{\left(2*NIR+1\right)}^{2}-8*\left(NIR-R\right)}}{2}$	Elimination of chlorophyll reflectance from soil background
SATVI	$\frac{SWIR_{1}-R}{SWIR_{1}+R+1}*2-\frac{SWIR_{2}}{2}$	Chlorophyll reflectance with the addition of short-wave infrared
SCI	(R−G)/(R+G)	Soil color
SRI	$R^{2}/\left(B*G^{3}\right)$	Hematite

Notes: $B,\;G,\;R,\;NIR,\;SWIR_{1},\;SWIR_{2}$ represent the B2, B4, B8, B11, B12 bands in Sentinel-2 images, respectively. MSAVI, modified soil-adjusted vegetation index; SATVI, soil-adjusted total vegetation index; SCI, soil color index; SRI, soil red index.

**Table 2:** Distance correlation coefficients between SOC and predictor variables.

Image Variables	B3	B11	RVI	DVI	NDVI
SOC_dcor	0.1879	0.2992	0.1645	0.0625	0.1511
EVI	SAVI	MSAVI	SATVI	SCI	SRI
0.0926	0.0901	0.1428	0.1440	0.1112	0.1788
**Terrain Variables**	**Elevation**	**CNBL**	**CND**	**CI**	**PlC**
SOC_dcor	0.0432	0.0110	0.1250	0.0976	0.0683
PrC	Slope	TWI	VD	RSP	LSF
0.0545	0.1092	0.1192	0.0705	0.1161	0.0258

Notes: SOC_dcor indicates the distance correlation coefficient between SOC and predictor variables. RVI, ratio vegetation index; DVI, difference vegetation index; NDVI, normalized difference vegetation index; EVI, enhanced vegetation index; SAVI, soil-adjusted vegetation index; MSAVI, modified soil-adjusted vegetation index; SATVI, soil-adjusted total vegetation index. SCI, soil color index; SRI, soil red index. CNBL, channel network base level; CND, channel network distance; CI, convergence index; PlC, plan curvature; PrC, profile curvature; TWI, topographic wetness index; VD, valley depth; RSP, relative slope position; LSF, length–slope factor.

**Table 3:** Descriptive statistics and normality test for SOC content (g/kg).

Data Set	Min	Max	Median	Mean	SE.mean
Total set	4.294	19.793	11.421	11.556	0.304
Calibration set	4.294	19.793	11.391	11.540	0.339
Validation set	6.676	18.408	11.480	11.632	0.697
**Data Set**	**Std.dec**	**Cope.var**	**Skewness**	**Kurtosis**	**Normative. *p***
Total set	3.261	0.282	0.119	−0.317	0.786
Calibration set	3.305	0.286	0.095	−0.277	0.775
Validation set	3.119	0.268	0.263	−0.903	0.757

**Table 4:** Comparison of the prediction accuracy among different models.

Models	Calibration			Validation		
	RMSEc	R^2^c	MAEc	RMSEv	R^2^v	MAEv
Ranger	**1.669**	**0.834**	**1.335**	***2.493***	***0.362***	***2.043***
Cubist	***1.88***	***0.693***	***1.518***	**2.283**	**0.445**	**1.877**
GBM	2.551	0.43	2.005	2.522	0.336	2.098
BRNN	2.712	0.323	2.121	2.589	0.282	2.058
RK-ranger	**1.553**	**0.867**	**1.194**	1.944	***0.718***	***1.604***
RK-Cubist	***1.646***	***0.794***	***1.256***	**1.741**	0.674	**1.474**
RK-GBM	2.050	0.734	1.556	***1.906***	**0.724**	1.608
RK-BRNN	2.369	0.569	1.797	2.066	0.625	1.632

Notes: the model with the highest accuracy is marked in bold; the model with the second highest accuracy is marked in bold italics.

## Data Availability

Not applicable.
